# Editorial: Improving the clinical value of digital phenotyping in mental health

**DOI:** 10.3389/fpsyt.2023.1251930

**Published:** 2023-07-18

**Authors:** Ang Li, Ronghui Liu, Xiaoqian Liu, Jin Han

**Affiliations:** ^1^Department of Psychology, Beijing Forestry University, Beijing, China; ^2^School of Economics and Management, University of Chinese Academy of Sciences, Beijing, China; ^3^Institute of Psychology, Chinese Academy of Sciences, Beijing, China; ^4^Black Dog Institute, University of New South Wales, Randwick, NSW, Australia

**Keywords:** digital phenotyping, mental health, artificial intelligence, machine learning, technology innovation

Reducing mental health problems and suffering is an integral part of the sustainable development goals. However, globally, the majority of those who need mental health care lack access to high-quality mental health services (1). Digital phenotyping refers to the collection and analysis of real-time biometric and personal data from digital devices (e.g., smartphones and wearable sensors) to generate moment-by-moment quantification of human function in health and disease (2), which has gained significant research and clinical interest. The clinical adoption of digital phenotyping in mental health care is expected to improve personalized diagnoses and treatment plans, and facilitate real-time monitoring of treatment effects and onset or relapse of mental illness (3).

Artificial intelligence (AI) and machine learning can be helpful tools in expanding digital phenotyping to improve the quality of mental health care. This Research Topic aims to introduce new advances in these areas. Four research articles, written by 20 influential researchers, were submitted and accepted by this Research Topic. These articles feature the new discoveries in digital phenotyping by using different types of biometric and personal data, including gait, voice, face, physical activity, and language expression.

Specifically, in the first article, Han et al. authored “*How social media expression can reveal personality*.” By analyzing linguistic characteristics of social media expressions, the authors proposed developing and testing machine learning models for predicting individual scores on the dimensions of Five-Factor personality, and demonstrated the importance of domain knowledge to the reliability, validity, and interpretability of model training.

In the second article, Huang et al. elaborated on “*Mental states and personality based on real-time physical activity and facial expression recognition*.” The authors collected data about facial motion and physical activity, and analyzed their correlations with the scores on questionnaires about personality and mental health. Results of this article highlighted the value of analyzing multimodal data as a means to improve the performance of digital phenotyping.

In the third article, Liu et al. wrote “*Ecological recognition of self-esteem leveraged by video-based gait*.” In this article, the authors focused on gait data acquired from more widely-used 2D camera rather than 3D camera, and built machine learning models to predict individual levels of self-esteem by their walking styles. Results of this article served as a good supplement to the existing methods for predicting self-esteem by gait analysis.

In the fourth article, Pan et al. investigated the differences in characteristics of vocal biomarkers between mentally-ill and healthy individuals. Their empirical study, “*Exploring the ability of vocal biomarkers in distinguishing depression from bipolar disorder, schizophrenia and healthy controls*,” supported the important role of vocal biomarkers in differentiating between people with and without mental illness.

The four above-mentioned articles provide valuable insights into ways to improve the clinical value of digital phenotyping in mental health care. Despite the fact that there is still a long way to go before the potential of digital phenotyping can be fully realized, we would like to express our appreciation to all authors, editors, and peer reviewers for their contributions to this Research Topic, and we look forward to continuing the dialogue and collaboration on this interesting topic.

## Author contributions

AL, RL, XL, and JH were guest editors for the Research Topic *Improving the clinical value of digital phenotyping in mental health* and co-authored the editorial. All authors contributed to the article and approved the submitted version.
